# Being Prepared During the Evolving COVID-19 Pandemic: A Neonatal Experience in Training and Simulation

**DOI:** 10.3389/fped.2021.785524

**Published:** 2021-12-02

**Authors:** Juin Yee Kong, Srabani Samanta Bharadwaj, Amutha Chinnadurai, Selina Kah Ying Ho

**Affiliations:** ^1^Department of Neonatology, KK Women's and Children's Hospital, Singapore, Singapore; ^2^Duke-NUS Medical School, Singapore, Singapore; ^3^Lee Kong Chian School of Medicine, Nanyang Technological University, Singapore, Singapore; ^4^Department of Pediatrics, Yong Loo Lin School of Medicine, National University of Singapore, Singapore, Singapore; ^5^Department of Neonatal and Developmental Medicine, Singapore General Hospital, Singapore, Singapore; ^6^Department of Neonatology, Khoo Teck Puat-National University Children's Medical Institute, National University Health System, Singapore, Singapore

**Keywords:** COVID-19, neonate, perinatal care, simulation, training

## Abstract

**Background:** Rapid spread of the COVID-19 pandemic raised an urgent need for preparedness in the healthcare sector, including training of healthcare workers to cope with the burden of infected cases while ensuring proper protection of themselves. Improper infection prevention and control measures were key reasons for infection in healthcare workers during the early phase of the outbreak.

**Objectives/Methods:** This paper describes the combined approach of 3 restructured hospitals in Singapore in preparing and training neonatal healthcare workers' during the COVID-19 pandemic crisis, as well as lessons learnt during this process.

**Results:** Information sharing was conducted in the form of e-learning, emphasizing on topics like disease knowledge and infection prevention and control procedures. Skills and competency training were carried out in the form of simulation, with sessions scaled into 4 levels progressing from individual task training to larger group simulations involving multiple disciplines and departments. Challenges encountered included information fatigue by large amount of constantly changing information and multiple amendments to workflows as more information arose. Difficulties conducting training and simulation sessions included restriction of group size to mitigate infection risk amongst participants and the limited supply of personal protective equipment prioritized for direct patient care.

**Conclusion:** Healthcare institutions should ensure adequate dissemination of conceptual knowledge as well as skills competency training of staff in infection control measures for the protection of healthcare workers and patient safety. Ongoing training for sustainability of knowledge and skills, while adapting to the rapidly evolving situation is important in the preparation for future outbreaks.

## Introduction

The first case of SARS-CoV-2 infection or Coronavirus Disease 2019 (COVID-19) was detected in Singapore on 23 January 2020 (1). By 30 January 2020, the WHO declared the novel coronavirus outbreak a Public Health Emergency of International Concern (PHEIC), and subsequently a global pandemic by 11 March 2020. A WHO guidance document released stressed that “all countries should increase their level of preparedness, alert and response to identify, manage and care for new cases of COVID-19” (2). The rapid spread of SARS-CoV-2 to Singapore and the rest of the world had resulted in a scramble in healthcare workers' (HCWs) training, in order to ensure proper protection while coping with the burden of infected cases (3–6). The urgency to complete this training, coupled with initial uncertainty regarding transmission risks and viral virulence contributed to the challenges in HCWs training. Lack of knowledge in IPC and insufficient time for systematic training and practice were some of the reasons for HCWs becoming infected with COVID-19 in China (4).

In Singapore, the lessons learnt from the 2003 Severe Acute Respiratory Syndrome (SARS) outbreak resulted in the public healthcare system having a higher level of preparedness against infectious disease threats (7). Post-SARS, many of the healthcare institutions strengthened IPC measures and developed systems, processes and infrastructure to manage infectious diseases and outbreaks. A 330-bed purpose-built National Centre for Infectious Diseases (NCID) was established in 2019 to strengthen the country's capabilities in infectious disease management and prevention. However, despite the many preparations and measures taken since 2003, there was still an urgent need for training and re-training due to the uncertainties about COVID-19 and its rapid spread.

Pregnant women and infants are especially vulnerable to COVID-19, due to the higher risk of morbidities and mortality in infected mothers, in addition to the early uncertainties regarding vertical and perinatal transmissions to their newborns (8). Implementing training on management of the mother-newborn dyad affected by COVID-19 was complicated due to the varied recommendations, and the need for inter-disciplinary inter-specialty collaboration (9–14). This article describes the combined approach of 3 restructured hospitals in Singapore: (a) KK Women's and Children's Hospital, (b) National University Hospital, and (c) Singapore General Hospital; in preparing and training neonatal HCWs during the COVID-19 pandemic, as well as lessons learnt during this process. These 3 hospitals provide maternity and neonatal services to an average of 47% of Singapore's annual live birth rate of ~39,000 in the last 3 years (15). As part of the country's response to the pandemic, all suspected/confirmed COVID-19 pregnant women were transferred to one of these 3 hospitals for triage and management.

## Training and Simulation

The emergence of the COVID-19 pandemic placed a huge strain on healthcare resources, and it was imperative to establish a rapid and efficient training programme. Training is more effective when HCWs understand the disease pattern, transmission risk and rationale for the recommendations. Goals should be focussed on patient care and HCW safety, ensuring that HCWs are well-prepared with adequate plans in different delivery scenarios. Training tools applied included e-learning, table-top exercises (talk-throughs), walk-throughs and real-time simulations including rapid cycle deliberate practice *in-situ* simulations (16). Adopting the quality improvement concept of “Going to the Gemba” approach for the walk-through was useful in observing the challenges faced at the real place [delivery room, operating theater (OT)] and has been used in pandemic preparedness (17).

### Information Sharing

Due to the need for safe distancing measures, e-learning has become an essential component of information sharing to avoid face-to-face contact. E-learning can be in the form of virtual presentations, interactive video conferencing with scenario-based discussions, or circulation of newsletters and infographics. Important topics to cover include hand hygiene technique, appropriate personal protective equipment (PPE) use (donning and doffing sequence), clinical care, and disinfection procedures. Display of posters and pictorial guides *in-situ* e.g., PPE guide at the entrance of patient's room can serve as reminders to HCWs. The fluid status and changing understanding of COVID-19 had resulted in frequent amendments to protocols and policies, which needed to be disseminated accurately and quickly via institutional online webpage, email and messaging groups amongst stakeholders.

### Simulations and Skills Competency Training

Simulation has been used and proven to be effective in previous outbreaks such as the SARS, Middle East Respiratory Syndrome-Corona virus (MERS Co-V), Ebola and Influenza (H1N1) (18–20). It plays a particularly important role when developing, teaching and implementing guidelines while identifying latent threats in newly developed processes and workflows (19–21). Its strength in education lies in providing practical application of knowledge and skills, and mirroring of different real-life situations without incurring risk to patients or compromising HCW safety (22). Simulation has been widely used to aid the training of HCWs in a timely manner, during the COVID-19 pandemic (23–34).

While HCWs routinely receive training in proper PPE use, studies have shown that its use is suboptimal, accompanied with high rates of error and contamination (21, 35). The efficacy and accuracy of donning and doffing of PPE, can be further enhanced by using simulation-based training (36, 37). Experience from the SARS and Ebola outbreaks showed a decrease risk of infection when training programs and adequate PPE were available (38–41). Simulated practice also affords psychological benefits to HCWs by improving confidence and relieving emotional stress (29, 39, 41–44). This is particularly relevant as fear, anxiety, and increased stress were reported among HCWs in Singapore during the SARS outbreak (45).

**Figure 1:**
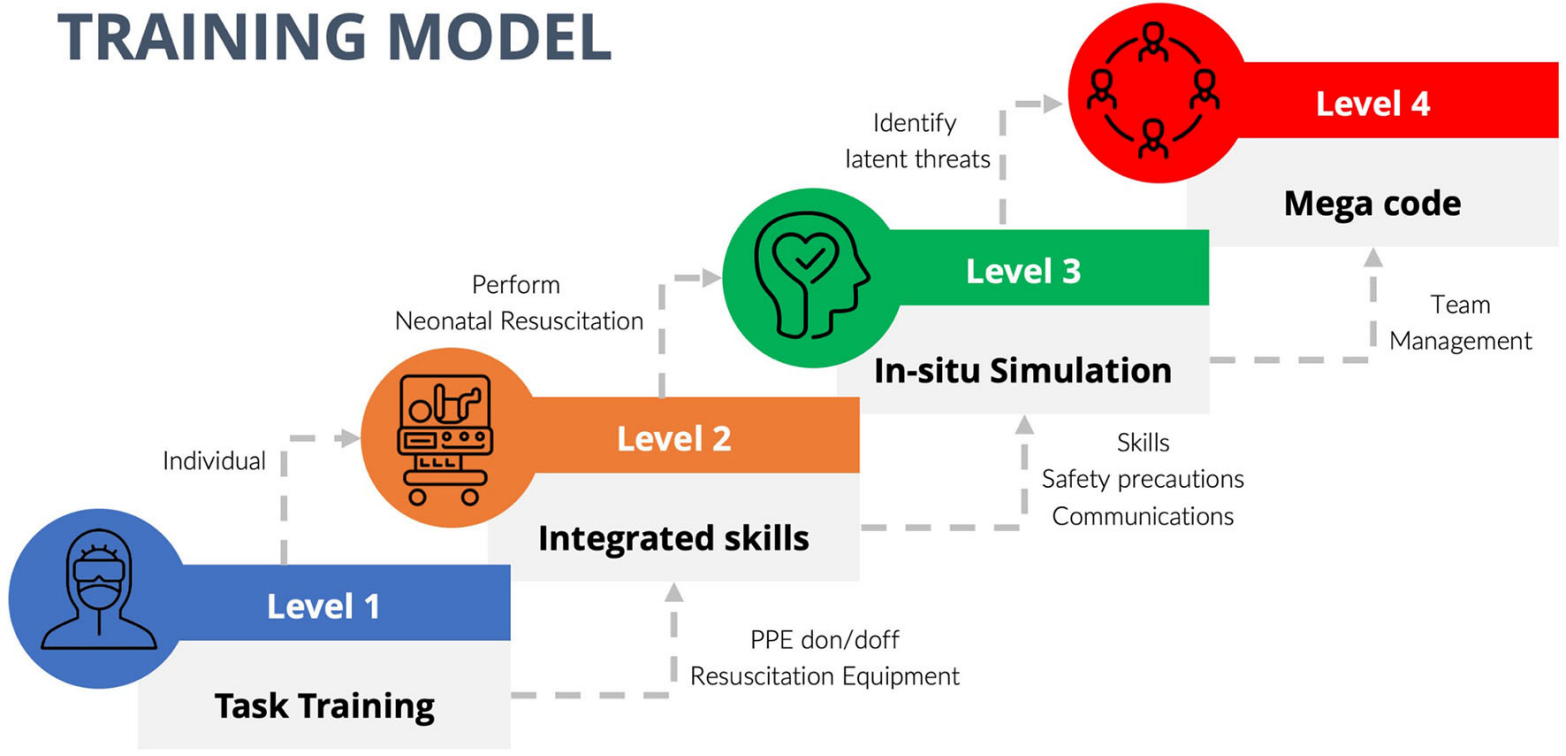
Structured training model with progressive levels.

In anticipation of the surge in disease volume, the plan for continuity of business in primary clinical areas and the resource allocation need to be taken into consideration when planning the simulation in each institution. The resources available in each center in terms of manpower, equipment, and patient delivery locations may require individual modification and adaptation. We propose that training is best delivered in progressive levels (Levels 1–4) directed at HCW's specific educational need with provision for adding other objectives as required within the structured framework (Figure 1). Overview of the different levels of training is summarized below:

#### Level 1—Individual Task Training

This is basic level training to ensure confidence and competency in using PPE and setting up of resuscitation equipment. The target participants were HCWs involved in neonatal resuscitation including nursing, midwifery, and medical personnel. IPC principles specific to COVID-19 were explained followed by interactive discussion and actual task training.

a) Proper donning and doffing of PPE: PPE are only protective when used correctly. The task was initially demonstrated by IPC officers, with subsequent practice by participants. Doffing of PPE has been perceived to be more complicated by staff than donning and identified as an area of ongoing confusion with risk of contamination (21). Training was enhanced using deliberate practice, with expert educators present reinforcing the donning and doffing, and placing sequential visual aids at all donning and doffing areas.
b) Resuscitation equipment set-up and disconnection: Participants were made conversant with the changes in workflow and safety principles, especially with regards to positive pressure ventilation (PPV) and aerosol generating procedures (AGPs). The use of High Efficiency Particulate Air (HEPA) filter on the PPV equipment (self-inflating bag and T-piece resuscitator) was demonstrated with participants having the opportunity to practice on task trainer manikins.

#### Level 2—Integrated Skills

Once competent with Level 1 training, participants proceeded to the next level of integrated skills training where they were required to perform the different steps of neonatal resuscitation while wearing full PPE (goggles, face shield, N95 masks or respirator, gown, gloves). Emphasis was placed not only on skills performance but also the safety precautions whilst performing AGPs, and the quality of communication between participants. This level addressed the difficulty of viewing the airway during intubation and challenges of interpersonal communication with the barrier of PPE. Target participants in the team included doctors, nurses and respiratory therapists (RTs). Examples of scenarios were (a) Performing PPV with PPE, (b) Performing intubation with/without surfactant administration with PPE, (c) communication and conveyance of information with PPE. This was conducted through low-fidelity, rapid-sequence simulation drills, using real equipment in the usual working environment (Supplementary Figure 1).

#### Level 3 –Intradepartmental *in-situ* Simulation

This level was aimed at familiarization of all staff involved in the admission of a newborn delivered to a suspected/confirmed COVID-19 mother and identifying any latent gaps in the existing workflow to ensure a seamless care process. The entire workflow was simulated starting from activation for delivery attendance to admission of the newborn to the Neonatal Unit. Personnel involved included doctors, nurses, RTs and admitting clerks. Some of the scenarios were (a) Term newborn with respiratory distress needing CPAP, (b) Term newborn born via meconium-stained amniotic fluid needing tracheal suctioning, (c) Extremely preterm newborn with respiratory distress syndrome, (d) Transfer of critically ill newborn to neonatal intensive care unit (Supplementary Figure 2), (e) Acute cardiorespiratory deterioration of an existing patient in the isolation unit requiring extensive resuscitation.

The facilitators encouraged participants to voice concerns and talk through problems with a view to identify latent threats and address issues (“Going to the Gemba”), and then devise solutions to overcome them (46). These were conducted at regular intervals as per duty roster and included the full team who will be involved. Many cognitive blind spots were identified at each simulation session and the workflow was amended accordingly.

#### Level 4—Multidisciplinary Interdepartmental Mega Code

This largest scale training was conducted as a multidisciplinary mega code which ran from activation and transfer of a suspected/confirmed COVID-19 mother, followed by delivery and resuscitation of the newborn, and finally the disposition of mother-newborn dyad post-delivery. The objective was to identify any gaps in communication and workflow, as well as to address technical and behavioral factors in the multidisciplinary setting. Simulation included pre-briefing, preparation of delivery area, post-delivery disposition of the mother-newborn dyad and disinfection of all involved areas (Supplementary Figure 3). Post-simulation debriefing was conducted to identify gaps in interdisciplinary communication, resource management and IPC measures. Departments involved included Neonatology, Obstetrics & Gynecology (OG), Infectious Disease, Security Services, Women's Anesthesia, Patient Transport Services, and Housekeeping Services. This type of simulation was repeated at less frequent intervals, mainly during change of staffing or if there were major changes in the workflow.

In order to ensure effective and constructive simulation, “spotters” were assigned to identify gaps and provide immediate feedback or take notes for discussion during post-simulation debriefing. Spotters were members outside of the immediate care providing team, e.g., IPC officer or patient safety lead whose focus was on the adherence of IPC measures including physical distancing while providing uncompromised patient care. When possible, they were instituted in actual case scenario as well to help remind staff with PPE donning and doffing which was identified as a challenge area.

An average of 4–6 training sessions per month (weekly level 1–2, monthly level 3–4) involving at least 50 HCWs per hospital have been conducted in the initial period from March to June 2020. Frequency of sessions varied thereafter depending on changeover of staff and patient load. E-learning and talk-throughs were conducted at least once every 3 month or when there were new orientations.

## Challenges Met and Lessons Learnt

Feedback and debriefing minutes which were collected from participants after the training sessions were summarized in Table 1.

**Table 1:** Challenges met and lessons learnt during simulation practices.

**Scenarios**	**Gaps identified**	**Lessons learnt**
**Preparation and standby**		
OG to neonatal team communication	• “ISO” case NOT emphasized • Inadequate handover of pertinent information impaired neonatal team preparation • Lack of preparation of items that needed to go into the isolation delivery room	• “Check in” at beginning of each shift on all ISO patients • Notification when new “ISO” case admitted • Mention of “ISO” during CODE announcement e.g., “CODE BLUE FOR ISO NEONATE IN OT 4”
Donning of PPE	• Unfamiliarity with location for PPE don/doff • Incorrect PPE donning (gown not tied, hair not neatly tucked in PAPR)	• Assignment of spotters • Mirror and infographics as reminders • Individual task training and simulation
Preparing and transporting isolette/incubator to delivery suite/OT	• Uncertainty on nursing waiting time outside isolation delivery room awaiting signal for transfer • Lack of coordination between team inside and outside isolation delivery room • Transport team went through the wrong path or door	• Use of cordless phone for better communication between rooms • Signage along typical path used for isolation cases • Simulation of transport
**Resuscitation**		
Adherence to IPC measures	• Position of maternal bed and resuscitaire at times not >2 meters • Risk of exposure of the back of neonatal team to mother's face if insufficient space and same room delivery	• Spotters to ensure adequacy of physical distancing • OG and Neonatal staff awareness of space constraint by rearranging bed/surgical trolleys • Drawing/labeling on floors as guide to positioning of bed and resuscitaire • Team members check each other to ensure gown covers back
Communication with full PPE	• Difficulty hearing other team members when PAPR is on • Difficulty in identifying personnel from other teams with PPE donned	• Get attention of team member before speaking, ensure eye contact • Label names and roles on chest or forehead for easy identification e.g., OG nurse, Neonatologist etc.
**Transportation process**		
Transfer of baby from open resuscitaire to an enclosed transport incubator	• Receiving team not completely prepared • Double door opened simultaneously resulting in loss in negative pressure and risk of contamination • Insufficient oxygen supply during transfer	• Cordless phone to communicate when ready and stable for transfer using closed loop communication technique • Handover ventilator settings for outside team to set up—confirm understanding using closed loop communication • To be vigilant and limit the repeated opening and closing of double doors leading to loss of negative pressure • Ensure oxygen availability during transfer—unplug from wall source as the last task before leaving
Communication before leaving delivery room	• Delay in transport due to late arrival of security officers to cordon off passage to public	• Activate security early (when called for delivery attendance) in order to cordon off area during transport
**Admission to the neonatal ward**		
Initial admission orders	• Unable to carry out orders and treatments promptly due to lack of equipment or in coordination amongst support staff • Repeated opening and closing of isolation room door to retrieve treatment items risking contamination	• 1 doctor to stay outside of isolation room to write orders • Quick update of plan of care with nurses before entering isolation room • Bring consumables needed when entering—Nurses can prepare special item (e.g., ice for bloods)
Procedures and Communication with full PPE	• Physiologic monitor alarms sounds not heard due to barrier from PAPR/PPE • Difficulty viewing with goggles due to fogging (e.g., during placement of fine aEEG leads, starting IV cannula or umbilical lines catheterization)	• Clinicians need to be cognizant of baby's vital signs at all times especially on PAPR when alarm monitors might not be heard clearly • Optimize alarm sound setting • Use visors/PAPR for prolonged procedures with less fogging • Communication with outside team using cordless phone or scribble board
Doffing of PPE	• Confusion with the doffing steps to be performed inside and outside the isolation room	• Familiarization with task training, simulation and placement of pictorial guide

*OG, Obstetrics and Gynecology; PPE, personal protective equipment; PAPR, powered air-purifying respirator; ISO, isolation; IPC, Infection prevention and control; aEEG, amplitude integrated electroencephalography*.

### Information Fatigue

Messaging fatigue and desensitization to information among HCWs as a result of overwhelming information load at the beginning of the outbreak, with subsequently large amount of emerging literature and updates have been described (47, 48). This could impact the ability of staff to recall the information or messages disseminated to them previously (49). Supervisors can facilitate staff learning by summarizing the large amount of data into key information in the form of infographics and posters, ensuring that training materials are clear and easily understood. Maintaining staff morale whilst instilling a sense of personal responsibility for self-learning are equally important in view of the anticipated protracted duration of the situation and the need for frequent modifications to protocols and policies.

### HCW Health and Psychological Risks

It is important that the training session does not become a source of infection transmission. Infection risk has been reported during COVID-19 pandemic simulation exercise and it is important for educators to ensure no participants have symptoms suggestive of acute respiratory infection (50). It is proposed to conduct training in a step-wise approach, increasing the level of proficiency and involvement to ensure better transition and effective learning in trainees while minimizing need for large group gathering. There should be adequate hand hygiene stations with strict hand hygiene practiced together with enhanced disinfection of training equipment and manikins. Psychological issues like depression, anxiety, somatization symptoms, insomnia are genuine concerns faced by HCWs (51, 52). Hence, during times of increased physical workload and psychological stress, training needs to be performed with sensitivity to the HCWs emotional states.

### Requirements for Safe Management Measures

Simulation of a newborn delivery usually involves a large multidisciplinary team comprising neonatal, obstetric, and even OT and anesthesia departments in the setting of cesarean section. It is extremely challenging to hold a realistic simulation practice session, mirroring an actual event, while adhering to capacity limits (53). Additional considerations while conducting training include registration for contact tracing, ensuring proper masking, limiting participant numbers, use of larger rooms for training and debrief to allow for distancing >2 m apart and live-video streaming to facilitate observation off-site (54). Video recording the sessions showing expert performance facilitates education to a greater audience and can be useful for future review and distribution with key debriefing points if face-to-face training is challenging. This may also aid in alleviating trainer fatigue when few educators need to conduct multiple sessions.

### Limited PPE and Equipment Supply

Realistic training and simulation mimicking real scenarios are critical for effective learning; however, this needs to be balanced with limited PPE and equipment supplies. Training amidst a worldwide shortage of PPE and equipment requires compromise and innovation. In order to conserve precious resources for direct patient care, training can include using expired or damaged equipment, utilizing visual representations and re-using or making mock PPE/equipment (29).

### Frequent Change in Guidelines

Meaningful scenarios have clearly defined and focussed objectives, with emphasis placed on incorporating updated knowledge and recommendations. Hence, the frequent guideline changes necessitated constant amendments to the simulation scenarios. To ensure proficiency and adaptation to new workflows, there is inevitable need for repeated simulation and retraining from time to time. Future scenarios can be based on real clinical encounters for more realistic representation of case scenarios.

### Lapse of Communication

Lapse of communication and identification of correct personnel was a real challenge between disciplines after donning PPE. Identifying personnel with labels e.g., “Neonatologist” and “Midwife” may help quick recognition of roles and improve communication. Having clear role allocation and rehearsing of communication techniques including non-verbal communication while maintaining closed loop communication can be useful. Clear instructions provided for access to equipment (bundling of items for ease of procedural preparation) and PPE (marking or labeling of specific areas designated for donning and doffing) can also reduce the need for repeated communication during the exercise.

## Discussion

In the early phases of the COVID-19 pandemic, HCWs were active in seeking training due to the evolving crises and urgency. However, sustaining clinician engagement in training has been a challenge, especially as routines resume, competing for the HCWs' time. To sustain this initial heightened desire for training and intra and interdepartmental collaboration, training champions can be identified and faculty development enhanced. Buy-in by stakeholders (clinicians, simulation, and IPC teams) with support from senior management are also necessary for sustainability.

With the protracted nature of the pandemic, ongoing training with iterative simulations are important. The long term goals should focus on skills retention and competency evaluation, which are vital to ensure quality of later performance. Ongoing training initiatives can include a combination of developing new training materials (e.g., cognitive aids, instructional videos), short follow-up training sessions, table-top exercises and simulations modified based on real case experiences and updated guidelines. Other retraining tools or methods, like mental training, using the mind as a simulator to mentally rehearse the movements of a task or operation, has been used as a supplementary tool in learning surgical skills (55, 56). Online modules should be easily accessible to staff at their convenience, to aid reinforcement of knowledge pertaining to disease updates, changes in policies, and guidelines.

Future training should progress beyond that of knowledge, skills, procedures and processes, and focus on the integration with other behavioral outcomes like teamwork, leadership and communication. Structured worksite practice involving individual task training and integrated skills training, can be peer led and performed by the on-call team members before the start of every shift. In-service simulated scenarios or tabletop exercises can be conducted less frequently (weekly to monthly) within the department to ensure smooth coordination between multidisciplinary personnel during resuscitation and transfer of patient. Grand mega codes involving interdepartmental stakeholders can take place quarterly to ensure optimal teamwork and communication between departments.

One major limitation of this current paper is the lack of prospective data collection with regards to the different domains of the training and formal assessment of clinical outcomes before and after training sessions. As the priority was to safely and effectively train and equip HCWs during the urgent phase of pandemic, proper measurement of clinical outcomes were not performed. To date, there has been no HCW transmission during training or while caring for COVID-19 positive patients.

## Conclusion

Institutions should ensure adequate training of staff in IPC measures for specific neonatal situations. The dissemination of conceptual knowledge as well as skills competency training in anticipation and preparation for the management of a newborn with exposure to COVID-19 will ensure optimal patient care and safety for staff. Training can be performed in progressive levels from individual tasks, integrated skills, intra-departmental *in-situ* simulation to multidisciplinary inter-departmental Mega Code, with debriefing after to identify gaps in processes. Ongoing training for sustainability of knowledge and skills, while adapting to the rapidly evolving situation is important. It is hoped that the training lessons learnt during this pandemic will allow us to be proactive and better prepare for future pandemics and unique infectious disease threats.

## Data Availability Statement

The original contributions presented in the study are included in the article/Supplementary Material, further inquiries can be directed to the corresponding authors.

## Author Contributions

JK: conceptualization, methodology, investigations, data curation, writing-original draft, writing-review and editing, visualization, and project administration. SB: writing—original draft, investigations, data curation, writing-review and editing, and visualization. AC: writing-original draft, investigations, data curation, and writing—review and editing. SH: conceptualization, writing-original draft, writing—review and editing, project administration, and supervision. All authors agree to be accountable for the content of the work and approve of the version to be published.

## Conflict of Interest

The authors declare that the research was conducted in the absence of any commercial or financial relationships that could be construed as a potential conflict of interest.

## Publisher's Note

All claims expressed in this article are solely those of the authors and do not necessarily represent those of their affiliated organizations, or those of the publisher, the editors and the reviewers. Any product that may be evaluated in this article, or claim that may be made by its manufacturer, is not guaranteed or endorsed by the publisher.

## Supplementary Material

Simulation Scenarios (1) Routine care in a term well baby, (2) Advanced resuscitation in a term sick baby, (3) Stable preterm baby requiring mask CPAP, (4) Resuscitation and transfer of a sick preterm baby.

Supplementary Figure 1 Providing mask PPV to newborn delivered by emergency cesarean section to a mother with COVID-19 with PAPR on (Level 2 training).

Supplementary Figure 2 Transporting newborn infant delivered to mother with suspected COVID-19 from operating theater to isolation neonatal ICU after emergency Cesarean section (Level 3 training).

Supplementary Figure 3 Admission and stabilization of intubated infant delivered to mother with suspected COVID-19 (Level 4 training).
