# Supplementary material for: Being Prepared During the Evolving COVID-19 Pandemic: A Neonatal Experience in Training and Simulation
Source: Front Pediatr. 2021 Dec 2;9:785524. doi: 10.3389/fped.2021.785524 (PMC8674782; doi:10.3389/fped.2021.785524)
Supplement: Simulation Scenarios — (1) Routine care in a term well baby, (2) Advanced resuscitation in a term sick baby, (3) Stable preterm baby requiring mask CPAP, (4) Resuscitation and transfer of a sick preterm baby. [file Data_Sheet_1.DOCX]

# **Scenario 1 - Routine Care in a Term Well Baby**

| **Field** | **Text** |
| --- | --- |
| Title | Woman admitted to isolation room for COVID-19 at 39 weeks’ gestation is in labour |
| Subtitle | Preparation – history, location of delivery, personnel, communication  Safe donning and doffing of PPE  Safe resuscitation and transfer with proper infection control measures |
| Overview tab |  |
| Simulation Type | Simulation |
| Simulation time | 15 minutes |
| Debriefing time | 30 minutes |
| Level | Basic |
| Patient Type | Woman with COVID-19 in labour |
| Target Groups | Neonatal resuscitation team |
| Summary | A 30-year-old G_1_P_0_ woman with COVID-19 was admitted 3 days ago to the isolation ward. She has mild fever and sore throat. Today, at 39 weeks’ gestation, her labour has progressed but the second stage is prolonged and a caesarean section is arranged. |
| Learning Objectives | *By end of Simulation participants should be able to*   - Apply the Neonatal inpatient COVID-19 workflow into practise - Understand the importance of information gathering for logistical purpose - Understand the difference in logistics of attending delivery in same room as mother or adjacent room - Apply the principles of COVID-19 precautions including appropriate PPE donning and doffing - Provide peer-to-peer feedback on PPE use in real time - Prepare equipment – only essential equipment, in disposable packs; inclusive of transport incubator. Proper disinfection and disposal of items. - Perform primary assessment of a patient – discussion and shared decision regarding DCC, SSC and post-delivery location of patient as per local guidelines - Resist suctioning in a vigorous newborn - Transfer process of baby – transport incubator (preferred if available) or open care resuscitaire - Maintain closed loop communication between team members inside and outside the delivery area |
| Educational information | Available |
| Why use this scenario? | This scenario addresses   - Information gathering needs in expected delivery of mother with COVID-19 - Proper donning of PPE by all healthcare members involved - Communication and coordination between teams with PPE – identify challenges - Awareness about resisting routine suctioning in a baby born vigorous as it is an aerosol-generating procedure (AGP) - Safe doffing and disposal of PPE - Safe transfer of baby – logistics involved |
| Prepare tab |  |
| Location | Neonatal skills training area – LW, OT, Neonatal unit or Simulation centre |
| Participants | Midwife, Obstetric nurse (ON), Neonatal Nurse (NN), attending Neonatologist, Neonatal trainees, |
| Preparation and setup | - Open care resuscitaire – self-contained with adequate gas cylinder and portable suction canister - Alcohol-based hand rub - Full PPE set - Shoe cover, Head cover, Goggles/face shield, N95 mask/PAPR, Long-sleeved, disposable gown, Gloves (long-sleeved if available) - PPE donning area as pre-identified for real-life situation - Essential equipment for resuscitation in individualised bags (institution based) - Disposable T-piece resuscitator and disposable BVM - HEPA filter, different sized masks, oxygen tubing - Stethoscope, suction catheter, towels, cap, SpO_2_ probe with self-adhering bandage - Equipment checklist - Term manikin - task trainer |
| Patient data | Mrs X 30 year old G_1_P_0_  Admitted in Isolation ward with COVID-19 |
| Simulate tab |  |
| Learner brief | *The” learner brief” should be read out loud to all the learners before the simulation starts.*  You are the neonatal trainee on-call  **Situation:** Called by LW nursing officer about caesarean section in OT  **Background:** 30 year old expectant woman has COVID-19. She was admitted 3 days ago with symptoms of fever and sore throat. She is in the isolation ward. She is at 39 weeks’ gestation, EFW 3.0kg. cardiotocography (CTG) reactive. G_1_P_0_ – GBS status negative, no prolonged rupture of membranes (PROM), no pyrexia. Spontaneous onset of labour.  **Assessment:** Her labour has progressed but the second stage is prolonged and a caesarean section is arranged. The CTG is reactive.  **Recommendation:** You are called for standby and to activate your COVID-19 team |
| Expected Interventions  (in accordance with local guidelines and can be amended accordingly) | - Activate Neonatal inpatient COVID-19 workflow – inform medical team and nurse in charge, prepare neonatal isolation room, inform on-duty attending Neonatologist & Neonatal fellow (if off site) - Standby team (minimum personnel) - Equipment to bring to standby area – full PPE [inclusive of N95 mask, eye protection (Goggles or Face shield), gown, gloves, cap & shoe cover], PAPR, essential equipment bags with relevant equipment, transport incubator (check tanks full) - Don full PPE – N95 in the donning area before entering OT and do peer to peer feedback - Placement of resuscitaire in an adjacent room or at least 2m away from mother (institution specific) - Role allocation of team members - Discussion and shared decision with OG team and expectant mother regarding DCC and SSC - Baby delivered by caesarean section under spinal anaesthesia – baby cried immediately, cord management per shared decision - Initial steps & Assessment – dried - Avoid routine suctioning in baby – assessment with routine care - Attach SpO2 probe (to facilitate monitoring during transfer) - Show baby to mother or birthing partner (if present) as per shared decision - Transfer baby to transport incubator - Communicate readiness of transfer to designated post-delivery location - Appropriate doffing – as per guidelines - Wear new PPE - Leave OT when signalled to do so by assistant or security. Check that baby remains stable before leaving - Transfer baby to separate neonatal unit or room-in with mother in full PPE (negative pressure room or designated isolation room) as per joint decision and clinical condition of the mother and baby.   **Critical steps:**  Peer to peer PPE check  Wear N95 at all times  Resist oro/naso-pharyngeal suctioning  Doffing procedure – depending on standby within mother’s room or in adjacent room  Safe disposal of equipment  Closed loop communication |
| Debrief tab |  |
| Debrief summary | - Summarize key learning points - Critical points that cannot be missed as mentioned in the critical steps section above - Identify any gaps and address lessons learnt and discuss possible solutions - Personal safety at all times - What would you do differently next time in a similar situation? - Take home messages |

Abbreviations: Labour Ward (LW), Operating Theatre (OT), SSC (Skin to skin Care), DCC (Delayed Cord Clamping), G (Gravida), P (para), NICU (Neonatal Intensive Care), PPE (Personal Protective Equipment), PAPR (Powered air-purifying respirator), HEPA (High Efficiency Particulate Air), SpO_2_ (oxygen saturation), GBS (Group B Streptococcus), OG (Obstetrics & Gynaecology), EFW (Estimated Foetal Weight); BVM (Bag Valve Mask)

# **Scenario 2 - Advanced Resuscitation in a Term Sick Baby**

| **Field** | **Text** |
| --- | --- |
| Title | A 40 year old woman in imminent labour arrives at A&E with a history of fever and cough for a day |
| Subtitle | Preparation – history, location of delivery, personnel, communication  Safe COVID19 specific measures in a Term baby needing advanced resuscitation  Safe transfer with proper IPC measures |
| Overview tab |  |
| Simulation Type | Simulation |
| Simulation time | 20 minutes |
| Debriefing time | 40 minutes |
| Level | Advanced |
| Patient Type | Woman with ARI symptoms in imminent labour |
| Target groups | Neonatal resuscitation team, OG team, A&E team |
| Summary | A 40 year old G_4_P_3_ lady presents to A&E with history of fever and cough for 1-day duration. She has a recent history of travel overseas and was on home quarantine for past 7days. She went into spontaneous labour today at 37 weeks and 2days gestation and presented to A&E in imminent labour with symptoms of ARI. |
| Learning Objectives | *By end of Simulation participants should be able to*   - Apply the Neonatal inpatient COVID-19 workflow into practise - Understand the importance of information gathering for logistical purpose – Mother with acute respiratory infection (ARI), positive travel history - Understand the difference in logistics of attending COVID19 woman’s delivery in A&E and variations involved - Apply the principles of COVID-19 precautions including appropriate PPE donning and doffing - Provide peer to peer feedback on PPE use in real time - Prepare equipment – only essential equipment, in disposable packs; inclusive of open care resuscitaire and transport incubator. Proper disinfection and disposal of items. - Perform a primary assessment of a patient – discussion and shared decision regarding DCC, SSC and post-delivery location of patient as per local guidelines - Commence resuscitation as per local Neonatal Resuscitation algorithm adapted to COVID specific scenarios. - Resist suctioning as AGP unless airway obstructed - Use HEPA filter for PPV, ensure tight seal (use 2-person seal where required) - If intubation needed – Video laryngoscope with disposable blade is preferred; Otherwise use disposable laryngeal blade (return used equipment into bio-hazard plastic bags to reduce contamination) - If Cardiac compression is needed – follow local Neonatal resuscitation Guidelines. - Transfer process of baby – transport incubator (preferred if available) or open care resuscitaire with adequate IPC measures - Maintain closed loop communication between team members inside and outside the delivery area |
| Educational information | Available |
| Why use this scenario? | This scenario addresses   - Gathering information needed in imminent delivery of woman with ARI and positive travel history - Awareness of activating COVID-19 pathway in cases with history suspicious of COVID-19 (ARI and positive travel history, but unknown COVID-19 status) - Understand the variations involved with delivery in A&E - Proper donning of PPE by all healthcare members involved - Communication and coordination between teams with PPE – identify challenges - Practise advanced resuscitation including CPR wearing full PPE – identify challenges - Safe doffing and disposal of PPE - Safe transfer of baby – logistics involved |
| Prepare tab |  |
| Location | Neonatal skills training area – Neonatal unit or Simulation Centre |
| Participants | Midwife, Obstetric nurse (ON), Neonatal Nurse (NN), attending Neonatologist, Neonatal trainees |
| Preparation and setup | - Open care resuscitaire – self-contained with adequate gas cylinder and portable suction canister - Alcohol-based hand rub - Full PPE set - Shoe cover, Head cover, Goggles/face shield, N95 mask, PAPR (if not N95 fitted), Long-sleeved, disposable gown, Gloves (long-sleeved if available) - PPE donning area as pre-identified for real-life situation - Essential equipment for resuscitation in individualised bags (institution based) - Disposable T-piece resuscitator and disposable BVM - HEPA filter, different sized masks, oxygen tubing - Stethoscope, suction catheter, towels, cap,SpO_2_ probe with self-adhering bandage - Advanced airway equipment – Laryngoscope with battery, disposable laryngeal blade, Endotracheal tube (ETT) different sizes, ET CO_2_ detector - Equipment checklist - Term manikin which could be intubated - task trainer |
| Patient data | Madam X 40 year old, G_4_P_3_  At the A&E bay |
| Simulate tab |  |
| Learner brief | *The learner brief should be read out loud to the learners before the simulation starts.*  You are the neonatal trainee on-call  **Situation:** Called by A&E nursing officer about a 40 year old pregnant lady presenting to A&E with symptoms of ARI, fully dilated and delivery is imminent. A&E nurse in-charge has informed OG team too.  **Background:** Madam X is 40 years old, G_4_P_3_. She has had an uneventful pregnancy so far. She recently travelled back from an overseas trip has been on home quarantine for past 7days. She started with a fever and cough 1 day ago. She did not see a GP as quarantining but took Paracetamol at home. She went into labour early this morning and so she came to A&E as she is experiencing strong contractions.  **Assessment:** She has been examined by OG trainee and she is fully dilated with head nearly crowning.  **Recommendation:** You are called for standby |
| Expected Interventions  (in accordance with local guidelines and can be amended accordingly) | - A pregnant lady with ARI in current pandemic situation - suspect COVID-19 and advise to take to Isolation area in A&E if not already done and start appropriate IPC measures - Activate Neonatal COVID-19 pathway – inform relevant Medical and Nursing in-charge, prepare neonatal isolation room, inform on-duty attending Neonatologist & Neonatal fellow - Standby Team (minimum personnel) to A&E – check COVID 19 workflow - Equipment to bring to AE standby area – full PPE [inclusive of N95 mask, eye protection (Goggles or Face shield), gown, gloves, cap & shoe cover], PAPR, essential equipment bags with relevant equipment, open care resuscitaire, transport incubator (check tanks full) - Don full PPE – N95 in the donning area in A&E and provide peer to peer feedback - Take essential equipment only to the standby area - Placement of resuscitaire as far away from mum as possible in the adjoining bay in A&E area (ideally at least 2m) (institution specific) - Role allocation of team members - Discussion and shared decision with OG team and expectant parents regarding DCC and SSC - Baby delivered by NVD – not crying or breathing, floppy - cord clamped immediately, brought to resuscitaire - Initial steps & Assessment – Dried, gentle suctioning if secretions blocking airway (consider using bulb suctioning). Avoid deep suctioning. - Assess breathing and HR - Commence face mask PPV within 1min using T-piece or BVM attached to HEPA filter if apnoeic/gasping or HR<60/min - Attach SpO_2_ probe - Call for extra help - Assess for effective ventilation and perform ventilation corrective steps if needed - Ensure adequate face mask seal – use 2-person technique if indicated - Reassess after 30sec of effective PPV – HR, RR, and SpO_2_. Consider early intubation --> most experienced person - Attach ETT to ET CO_2_ detector and HEPA filter and commence ETT-PPV via T-piece or BVM, Titrate FiO_2_ - Reassess after 30sec – chest rise seen, HR <60/min, apnoeic, SaO_2_ 40% - Increase FiO_2_ to 100% (if not done already) --> Start coordinated Chest compression with ventilation as per local guidelines - Reassess after 1min – HR >100, SaO_2_ 92% in 100% FiO_2_ - Continue stabilisation – remember thermoregulation, titrate FiO_2_ according to SpO_2_ and check airway - Decision to transfer baby to transport incubator once ready – **attach HEPA filter to expiratory limb of transport incubator placed within incubator** if possible - Return used equipment into sealed plastic bags for disposal/decontamination - Communicate readiness of transfer to neonatal unit - Inform family about baby’s resuscitation and decision to admit to neonatal unit irrespective of initial agreement - Appropriate doffing – as per guidelines - Wear new PPE - Leave A&E when signalled to do so by assistant or security. Check baby’s clinical stability before leaving - Transfer baby to neonatal unit in full PPE (negative pressure room or designated isolation room)   **Critical steps:**  Standby in same area as delivery  Mother with ARI – full PPE necessary for management of babies born to mother suspected to have COVID-19 (even if mother not tested yet)  Peer to peer PPE check  Wear N95 at all times  Use HEPA filter with T-piece or BVM  Doffing appropriately  Safe disposal of equipment  Closed loop communication |
| Debrief tab |  |
| Debrief summary | - Summarize key learning points - Critical points that cannot be missed as mentioned in the critical steps section above - Personal safety at all times - Identify any gaps and address lessons learnt and discuss possible solutions - What would you do differently next time in a similar situation? - Take home messages |

Abbreviations: A&E (Accident & Emergency); IPC (Infection Prevention & Control); ARI (Acute Respiratory Infection); LW (Labour Ward); OT (Operating Theatre); SSC (Skin to skin Care), DCC (Delayed Cord Clamping), G (Gravida), P (para), NICU (Neonatal Intensive Care), PPE (Personal Protective Equipment), PAPR (Powered air-purifying respirator), HEPA (High Efficiency Particulate Air), SpO_2_ (oxygen saturation), GBS (Group B Streptococcus), OG (Obstetrics & Gynaecology), EFW (Estimated Foetal Weight); CPR (Cardiopulmonary Resuscitation); ETT (Endotracheal Tube); ET CO_2_ (End Tidal carbon dioxide); BVM (Bag Valve Mask); FiO_2_ (Fraction of inspired oxygen); NVD (normal vaginal delivery)

# **Scenario 3 - Stable Preterm Baby requiring Mask CPAP**

| **Field** | **Text** |
| --- | --- |
| Title | Woman with COVID-19 in preterm labour at 31 weeks’ gestation |
| Subtitle | Preparation – history, location of delivery, personnel, communication  Safe COVID-19 specific resuscitation of preterm baby  Safe transfer of preterm baby with proper IPC measures |
| Overview tab |  |
| Simulation Type | Simulation |
| Simulation time | 15 minutes |
| Debriefing time | 30 minutes |
| Level | Basic Level 2 |
| Patient Type | Labouring woman with COVID-19 |
| Target groups | Neonatal resuscitation team |
| Summary | A 25 year old G_1_P_0_ woman was diagnosed with COVID-19 when she presented with symptoms of fever, cough and sore throat 5 days ago and was admitted to Isolation ward. She has gone into preterm labour and admitted to the delivery room and labour is progressing. |
| Expected Task | *By end of Simulation participants should be able to*   - Apply the Neonatal inpatient COVID-19 workflow into practise - Understand the importance of information gathering for logistical purpose and variations involved – specific to preterm delivery. E.g. ANC, Magnesium sulphate - Apply the principles of COVID-19 precautions including appropriate PPE donning & doffing, proper disinfection processes and disposal of items. - Provide peer to peer feedback on PPE use in real time - Prepare equipment – All essential equipment, in disposable packs; including HEPA filter. Keep in readiness an open care resuscitaire and transport incubator - Perform a primary assessment of a patient – discussion and shared decision regarding DCC, SSC - Follow local Neonatal Resuscitation algorithm for specific steps to stabilisation and resuscitation of preterm newborn - Follow critical variations to routine steps adapted to COVID specific scenarios (resist routine suctioning, avoid prong CPAP, use HEPA filter) - Use HEPA filter for PPV, ensure tight seal (use 2-person seal where required) and also expiratory in the limb of CPAP during transfer back - Transfer baby following COVID specific precautions– transport incubator (if available) or open care resuscitaire with adequate IPC measures - Maintain closed loop communication between team members inside and outside the delivery area |
| Educational information | Available |
| Why use this scenario? | This scenario addresses   - Gathering relevant information needed in imminent delivery of COVID19 positive woman in preterm labour - Proper donning of PPE by all healthcare members involved - Communication and coordination between teams with PPE – identify challenges - Awareness about resisting routine suctioning in a baby born vigorous as it is an AGP - Safe application of mask CPAP and avoid nasal prong CPAP - Safe doffing and disposal of PPE - Safe transfer of baby – logistics involved with regards to CPAP during transit |
| Prepare tab |  |
| Location | Delivery room - resuscitation within the room or Simulation Centre |
| Participants | Midwife, Obstetric nurse (ON), Neonatal Nurse (NN), attending Neonatologist, Neonatal trainees |
| Preparation and setup | - Open care resuscitaire – self-contained with adequate gas cylinder and portable suction canister with HEPA filter - Alcohol-based hand rub - Full PPE set - Shoe cover, Head cover, Goggles/face shield, N95 mask, PAPR (if not N95 fitted), Long-sleeved, disposable gown, Gloves (long-sleeved if available) - PPE donning area as pre-identified for real-life situation - Essential equipment for resuscitation in individualised bags (institution based) - Plastic bag, disposable exothermic mattress - Disposable T-piece resuscitator and disposable BVM - HEPA filter, different sized masks, oxygen tubing - Stethoscope, suction catheter, plastic wrap, cap, SpO_2_ probe with self-adhering bandage - Equipment checklist - Preterm manikin - task trainer, role labels/markers as identifiers on PPE - Transfer equipment - transport incubator or giraffe shuttle (Institution specific) |
| Patient Data | Madam X, 25year old woman, primigravida. ARI symptoms 1week back. COVID-19 +ve. Admitted to isolation ward. |
| Simulate tab |  |
| Learner Brief | *The learner brief should be read out loud to the learners before the simulation starts.*  You are the neonatal trainee on-call in neonatal unit  **Situation:** Called by LW nurse in-charge to come for standby for preterm labour at 31wks GA about to deliver by NVD  **Background:** Madam X has COVID-19 and is at 31 weeks’ gestation. She was admitted 5 days ago with symptoms of ARI (fever, cough and sore throat). EFW 1.2 kg. CTG has been reactive. She is G_1_P_0_ with no GBS colonisation, no PROM. Spontaneous onset of labour.  **Assessment:** Her labour has progressed with strong contractions and VE shows OS 8 cm dilated. She has regular contractions of 3:1. The CTG is reactive.  **Recommendation:** You are called for standby and to activate your COVID-19 team |
| Expected interventions  (in accordance to local guidelines and can be amended accordingly) | - Activate Neonatal COVID-19 pathway – inform relevant Medical and Nursing personnel in-charge, prepare neonatal isolation room, inform on-duty attending Neonatologist & Neonatal fellow - Assign standby team (minimum personnel) - Don full PPE – N95 in the donning area and do peer to peer feedback - take essential equipment only to the standby area - COVID-19 specific equipment - Plan area of resuscitation - placement of resuscitaire in adjacent room or at least 2m away from mother (institution specific) - Role allocation of team members - Discussion and shared decision with OG team and expectant parents regarding DCC and SSC - Baby delivered by NVD – cries immediately, cord management as per shared decision - Initial steps & Assessment – plastic bag/cling wrap +/- exothermic mattress - Avoid routine suctioning in baby – assessment with routine care - Attach SpO_2_ probe - Assess vitals – If baby warrants respiratory support but has spontaneous respiratory effort, apply Mask CPAP with HEPA filter through T-piece, check temperature, ensure baby’s vitals are stable, Fio2 titrated to Saturations, get ready for transfer - Show baby to mother or birthing partner (if present) as per shared decision - Inform family about baby’s resuscitation and decision to admit to neonatal unit irrespective of initial agreement - Communicate readiness of transfer to neonatal unit; inform team outside re: CPAP – to **attach HEPA filter to expiratory limb of transport incubator placed within incubator** if possible - Return used equipment into sealed plastic bags for disposal/decontamination - Appropriate doffing – as per guidelines - Wear new PPE - Leave LW when signalled to do so by assistant or security. Check baby’s clinical stability before leaving; Ensure delivery of PEEP and baby’s saturation and HR are stable at all times - Transfer baby to neonatal unit in full PPE (negative pressure room or designated isolation room)   **Critical steps:**  Peer to peer PPE check  Standby maybe in the same room or adjacent room – discuss variations  Wear N95 at all times  Resist oro/naso-pharyngeal suctioning (AGP)  Use HEPA filter with T-piece, BVM and in expiratory limb of CPAP during transport  Avoid prong CPAP  Low threshold for intubation  Doffing appropriately - depending on standby within mother’s room or in adjacent room  Safe disposal of equipment  Closed loop communication |
| Debrief tab |  |
| Debrief summary | - Summarize key learning points - Critical points that cannot be missed as mentioned in the critical steps section above - Personal safety at all times - Identify any gaps and address lessons learnt and discuss possible solutions - What can be done differently next time in a similar situation? - Take home messages |

Abbreviations: ANC (Antenatal Corticosteroids) ; AGP (Aerosol generating procedure); CPAP (continuous positive airway pressure) IPC (Infection Prevention & Control); ARI (Acute Respiratory Infection); LW (Labour Ward); OT (Operating Theatre); SSC (Skin to skin Care), DCC (Delayed Cord Clamping), G (Gravida), P (para), NICU (Neonatal Intensive Care), PPE (Personal Protective Equipment), PAPR (Powered air-purifying respirator), HEPA (High Efficiency Particulate Air), GBS (Group B Streptococcus), OG (Obstetrics & Gynaecology), EFW (Estimated Foetal Weight); BVM (Bag Valve Mask); FiO_2_ (Fraction of inspired oxygen)

# **Scenario 4 – Resuscitation and Transfer of a Sick Preterm Baby**

| **Field** | **Text** |
| --- | --- |
| Title | Woman with COVID-19 admitted at 29 weeks’ gestation in labour with PV bleeding |
| Subtitle | Preparation – history, location of delivery, personnel, communication  Safe COVID-19 specific resuscitation of preterm baby  Safe transfer of preterm baby with proper IPC measures |
| Overview tab |  |
| Simulation Type | Simulation |
| Simulation time | 20 minutes |
| Debriefing time | 40 minutes |
| Level | Advanced Level 2 |
| Patient Type | Labouring woman with COVID-19 and bleeding PV |
| Target groups | Neonatal resuscitation team |
| Summary | A 25 year old G_4_P_3_ pregnant woman, was diagnosed with COVID-19 one day ago when she presented in early labour with symptoms of ARI (fever, cough and sore throat) for 3 days. The labour progressed and she was shifted to delivery ward this morning. She was then noted to have sudden PV bleeding and suspected abruptio placentae with poorly reactive CTG. She is activated for emergency caesarean section. |
| Expected Task | *By end of Simulation participants should be able to*   - Apply the Neonatal inpatient COVID-19 workflow into practise - Understand the importance of information gathering for logistical purpose and variations involved – specific to preterm delivery. E.g. ANC, Magnesium sulphate, APH - Apply the principles of COVID-19 precautions including appropriate PPE donning & doffing - Provide peer to peer feedback on PPE use in real time - Prepare equipment – only essential equipment, in disposable packs. inclusive of open care resuscitaire and transport incubator, proper disinfection and disposal of items. - Perform a primary assessment of a patient – discussion re: regarding DCC, SSC in the background of APH - Follow local Neonatal Resuscitation algorithm for specific steps to stabilisation and resuscitation of preterm newborn - Follow critical variations to routine steps adapted to COVID specific scenarios (resist routine suctioning, avoid prong CPAP, use HEPA filter, use Video laryngoscope with disposable blades if available or disposable laryngeal blade for direct laryngoscopy) - Use HEPA filter for PPV, ensure tight seal (use 2-person seal where required) - Low threshold for intubation – by most experienced person and ideally first pass - If Intubated, disposable laryngeal blade to be kept back inside plastic bags to reduce contamination - If CPR needed – follow local neonatal resuscitation algorithm but ensure adequate and effective ventilation prior to chest compression - Transfer process of baby – transport incubator (if available) or open care resuscitaire with adequate IPC measures - Maintain closed loop communication between team members inside and outside the delivery area |
| Educational information | Available |
| Why use this scenario? | This scenario addresses   - Gathering relevant information needed in imminent delivery of COVID19 positive woman in preterm labour with APH and abnormal CTG - Proper donning of PPE by all healthcare members involved - Communication and coordination between teams with PPE – identify challenges - Familiarize communication wearing PAPR and practice advanced resuscitation including CPR wearing full PPE in a preterm infant - Prepare for unexpected needs of things and manpower - Safe doffing and disposal of PPE - Safe transfer of baby – logistics involved with regards to CPAP during transit |
| Prepare tab |  |
| Location | Operating theatre - resuscitation within the room |
| Participants | Midwife, Obstetric nurse (ON), Neonatal Nurse (NN), attending Neonatologist, Neonatal trainees |
| Preparation and setup | - Open care resuscitaire – self-contained with adequate gas cylinder and portable suction canister - Alcohol-based hand rub - Full PPE set - Shoe cover, Head cover, Goggles/face shield, N95 mask, PAPR (if not N95 fitted), Long-sleeved, disposable gown, Gloves (long-sleeved if available) - PPE donning area as pre-identified for real-life situation - Essential equipment for resuscitation in individualised bags (institution based) - Plastic bag, disposable exothermic mattress - Disposable T-piece resuscitator and disposable BVM - HEPA filter, different sized masks, oxygen tubing - Stethoscope, suction catheter, plastic wrap, cap, SpO_2_ probe with self-adhering bandage - Advanced airway equipment – Laryngoscope with battery, disposable laryngeal blade, ETT different sizes, ETCO_2_ - Equipment checklist - Preterm manikin which could be intubated - task trainer - Transfer equipment - transport incubator or giraffe shuttle (Institution specific) |
| Patient Data | - Madam X is 25yr old woman, G_4_P_3_ with ARI symptoms 1 day prior to admission. Tested to be Covid +ve. Admitted to isolation ward. |
| Simulate tab |  |
| Learner Brief | *The learner brief should be read out loud to the learners before the simulation starts.*  You are the on-call neonatal trainee  **Situation:** Called by LW nurse about the activation of LSCS for preterm labour at 29 weeks GA with sudden onset of PV bleed. CTG shows poor variability.  **Background:** Madam X is 25 years old, G_4_P_3_ at 29 weeks of pregnancy. She has had an uneventful pregnancy so far. She started with fever, cough and sore throat 3 days ago. She was diagnosed to have COVID-19 yesterday when she presented in early labour. The labour progressed and she was shifted to delivery ward this morning. She developed sudden PV bleeding 15 minutes ago and suspected abruptio placentae with poor CTG.  **Assessment:** She has been examined by OG registrar and an emergency LSCS has been activated. OG consultant is arriving, and anaesthetist and OT have been informed.  **Recommendation:** You are called for standby and to activate your COVID-19 team |
| Expected Interventions  (in accordance to local guidelines and can be amended accordingly) | - Activate Neonatal COVID-19 pathway – inform relevant Medical and Nursing personnel in-charge, prepare neonatal isolation room, inform on-duty attending Neonatologist & Neonatal fellow - Assign standby team (minimum personnel) - Don full PPE – N95 in the donning area in OT and do peer to peer feedback - Check and take essential equipment only to the standby area - COVID-19 specific equipment - Plan area of resuscitation - placement of resuscitaire in adjacent room or at-least 2m away from mother (institution specific) - Role allocation of team members - Discussion and shared decision with OG team and expectant parents regarding contraindications to DCC and also critical nature of case - Perform equipment check, ensure HEPA filter is connected, identify working whether walkie talkie functional to communicate with team outside for information and call for additional help - Baby delivered by LSCS – appears pale, hypotonic, apnoeic, HR - 40/min - Commence PPV attached with HEPA filter Attach SpO_2_ probe - Assess for effective ventilation and perform ventilation corrective steps if needed; ensure adequate face mask seal – use 2-person technique if indicated - Reassess after 30sec of effective PPV – HR, RR, and SpO_2_ - Low threshold to intubate by most experienced person – use disposable blades / Video laryngoscope to minimize risk to resuscitation team - Attach ETT to ET CO_2_ detector and HEPA filter and commence ETT-PPV via T-piece or BVM, Titrate FiO_2_ - Continue stabilisation – remember thermoregulation, titrate FiO_2_ according to SpO_2_ and check airway - Ensure ETT in place and baby’s saturation and HR are stable at all times - Decision to transfer baby to transport incubator once ready – **attach HEPA filter to expiratory limb of transport incubator placed within incubator** if possible - Return used equipment into sealed plastic bags for disposal/decontamination - Communicate readiness of transfer to neonatal unit including sick status of baby - Inform family about baby’s resuscitation and decision to admit to neonatal unit irrespective of initial agreement - Way cleared by security and transfer begins - Registrar and RN doffs - shoe cover, gloves, hand rub, yellow gown, hand rub and go out with PAPR. Don PPE again outside the room and follow the team   **Critical steps:**  Peer to peer PPE check  Standby maybe in the same room or adjacent room – discuss variations  Wear N95 at all times  Resist oro/naso-pharyngeal suctioning  Remember thermoregulation  Use HEPA filter with T-piece, BVM and in expiratory limb of ventilator circuit during transport  Low threshold for intubation -  Doffing appropriately - depending on standby within mother’s room or in adjacent room  Safe disposal of equipment  Closed loop communication |
|  |  |
| Debrief tab |  |
| Debrief summary | - Summarize key learning points - Critical points that cannot be missed as mentioned in the critical steps section above - Time taken overall and reason for delays - Identify any gaps and address lessons learnt and discuss possible solutions - What can be done differently next time in a similar situation - Personal safety at all times - Take home messages |

Abbreviations: APH (Antepartum Haemorrhage); ANC (Antenatal Corticosteroids) ; AGP (Aerosol generating procedure); CPAP (continuous positive airway pressure) IPC (Infection Prevention & Control); ARI (Acute Respiratory Infection); LW (Labour Ward); OT (Operating Theatre); SSC (Skin to skin Care), DCC (Delayed Cord Clamping), G (Gravida), P (para), NICU (Neonatal Intensive Care), PPE (Personal Protective Equipment), PAPR (Powered air-purifying respirator), HEPA (High Efficiency Particulate Air), SpO_2_ (oxygen saturation), GBS (Group B Streptococcus), OG (Obstetrics & Gynaecology), EFW (Estimated Foetal Weight); BVM (Bag Valve Mask); FiO_2_ (Fraction of inspired oxygen); LSCS (lower section caesarean section)
