# Supplementary figures and images for: Being Prepared During the Evolving COVID-19 Pandemic: A Neonatal Experience in Training and Simulation
Source: Front Pediatr. 2021 Dec 2;9:785524. doi: 10.3389/fped.2021.785524 (PMC8674782; doi:10.3389/fped.2021.785524)

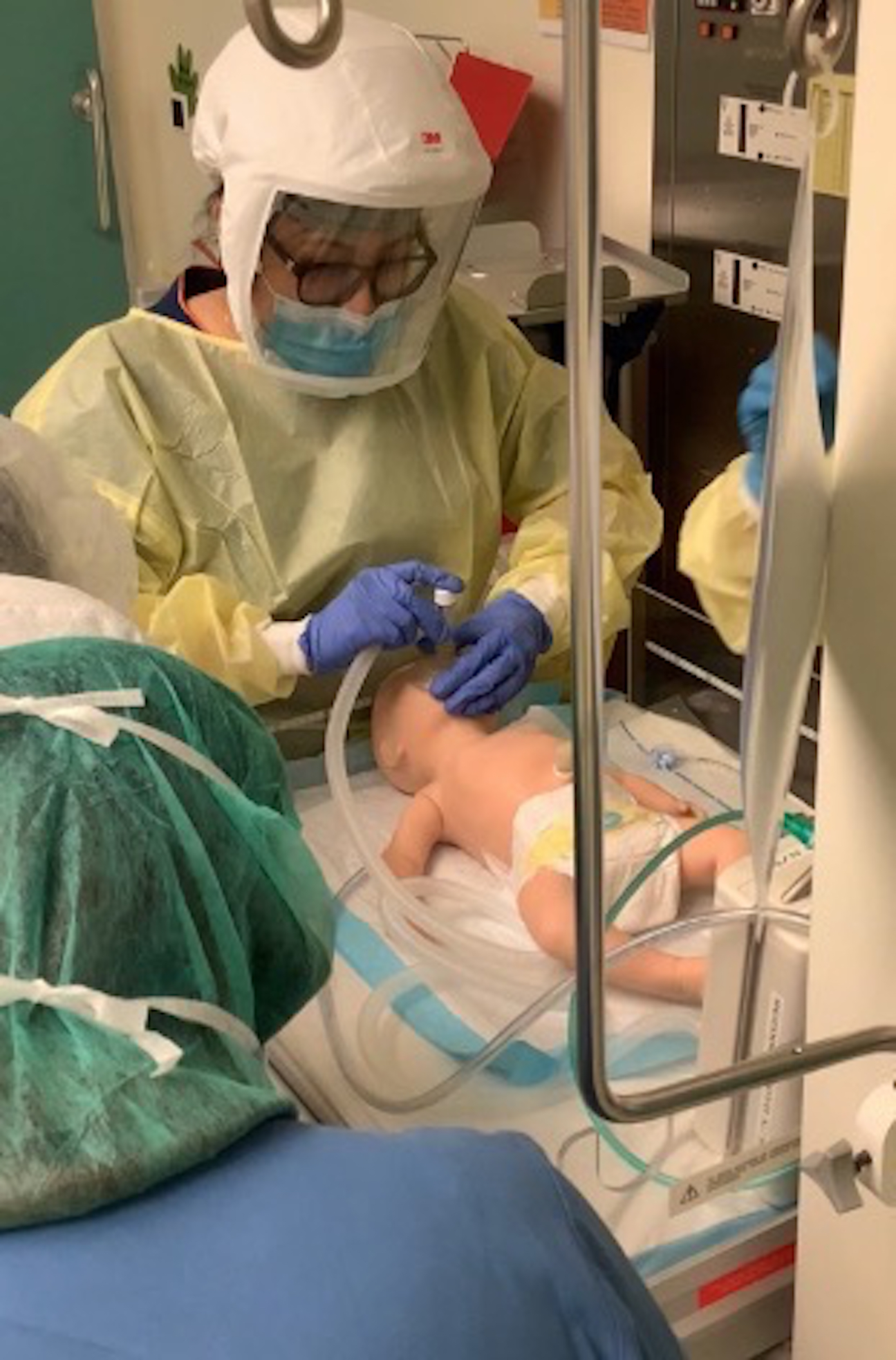

Supplement: Supplementary Figure 1 — Providing mask PPV to newborn delivered by emergency cesarean section to a mother with COVID-19 with PAPR on (Level 2 training). [file Image_1.JPEG]

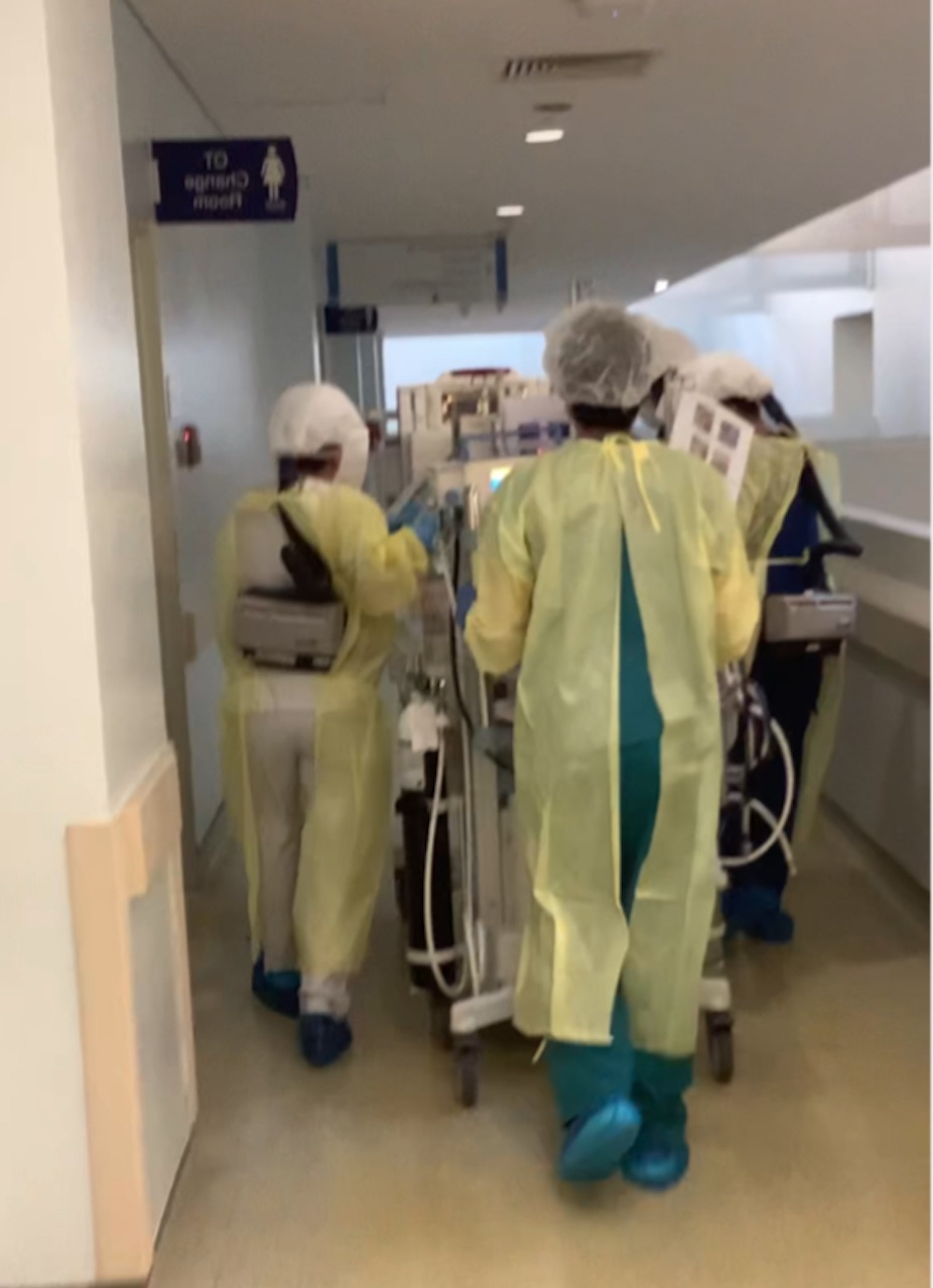

Supplement: Supplementary Figure 2 — Transporting newborn infant delivered to mother with suspected COVID-19 from operating theater to isolation neonatal ICU after emergency Cesarean section (Level 3 training). [file Image_2.JPEG]

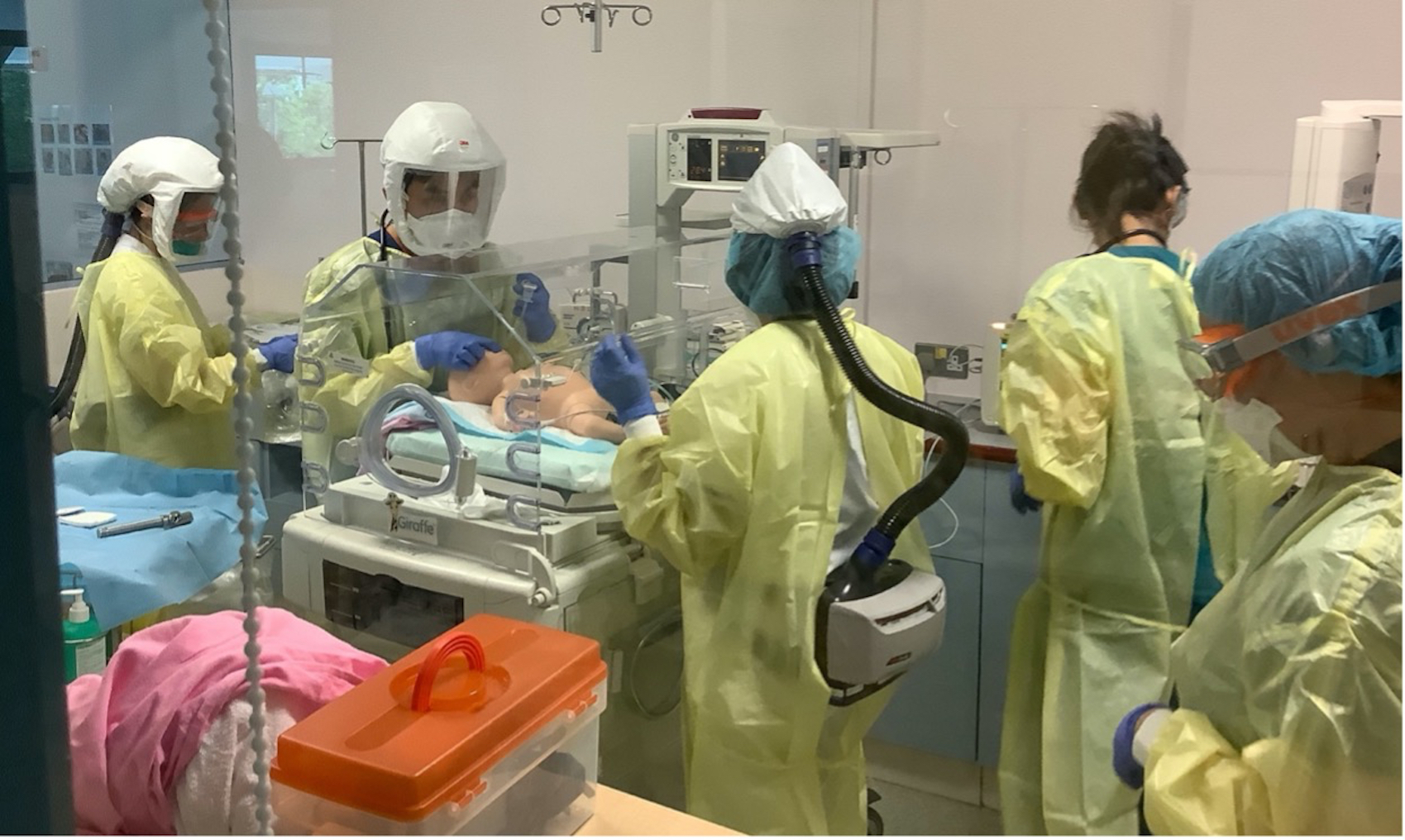

Supplement: Supplementary Figure 3 — Admission and stabilization of intubated infant delivered to mother with suspected COVID-19 (Level 4 training). [file Image_3.JPEG]
